# iFIND INH/FQ: a LC-aNAAT assay for rapid simultaneous detection of isoniazid and fluoroquinolone resistance in *Mycobacterium tuberculosis*

**DOI:** 10.1128/spectrum.03743-25

**Published:** 2026-02-06

**Authors:** Xichao Ou, Yingzi Ma, Huiwen Zheng, Yan Li, Jiaojian Zeng, Yuying Chen, Lin Huang, Yajie Guo, Bing Zhao, Jingjing Li, Ruida Xing, Hui Xia, Yanlin Zhao

**Affiliations:** 1 National Key Laboratory of Intelligent Tracking and Forecasting for Infectious Diseases, National Center for Tuberculosis Control and Prevention, Chinese Center for Disease Control and Prevention (Chinese Academy of Preventive Medicine) 12415, Beijing, China; 2 Tuberculosis Prevention and Control Division, Chengde Center for Disease Control and Prevention (Chengde Health Inspection Institute), Chengde, China; 3 Beijing Key Laboratory of Pediatric Respiratory Infection Diseases, Key Laboratory of Major Diseases in Children, Ministry of Education, National Clinical Research Center for Respiratory Diseases, Laboratory of Respiratory Diseases, Beijing Pediatric Research Institute, Beijing Children’s Hospital, Capital Medical University, National Center for Children’s Health 12517, Beijing, China; Boston Medical Center, Boston, Massachusetts, USA

**Keywords:** *Mycobacterium tuberculosis*, drug resistance, isoniazid, fluoroquinolones, molecular diagnostics

## Abstract

To evaluate the performance of the iFIND INH/FQ, a low-complexity molecular assay, for the rapid and simultaneous detection of resistance to isoniazid (INH) and fluoroquinolones (FQs) in *Mycobacterium tuberculosis*. Frozen sputum specimens stored at the Chengde Center for Disease Control and Prevention laboratory were used. Phenotypic drug susceptibility testing (pDST) and DNA sequencing served as reference standards. The limit of detection (LOD) was determined using probit regression with spiked samples. The assay’s ability to detect resistance-conferring mutations in *katG*, *inhA*, and *gyrA* genes was assessed using genotypically characterized strains. Diagnostic accuracy was evaluated against pDST. The LOD was 20.79 CFU/mL for INH and 9.34 CFU/mL for FQs. The assay detected all targeted mutations except *ahpC* c.-6 associated with INH resistance. Compared to pDST, the iFIND INH/FQ assay demonstrated a sensitivity of 97.59% (95% confidence interval [CI]: 91.63–99.34%) and specificity of 98.10% (95% CI: 94.57–99.35%) for INH resistance. For FQ resistance, sensitivity was 92.16% (95% CI: 81.50–96.91%) for levofloxacin and 92.00% (95% CI: 81.16–96.85%) for moxifloxacin, with specificities of 97.33% (95% CI: 93.89–98.85%) and 96.81% (95% CI: 93.21–98.53%), respectively. Sequencing confirmed iFIND results in the majority of discrepant cases (100% for INH and 55.65% for FQs). The iFIND INH/FQ LC-aNAAT is a highly accurate and rapid molecular assay for simultaneous detection of INH and FQ resistance. It is a promising tool for scaling up rapid drug susceptibility testing in clinical and peripheral laboratory settings.

**IMPORTANCE:**

As a low-complexity automated nucleic acid amplification test, the iFIND assay achieves the goal of simultaneously detecting isoniazid and fluoroquinolone resistance in approximately 90 min, perfectly meeting the TPP's core requirements for “rapid” and “simple operation.” Its fully integrated system minimizes manual steps and contamination risk, making it highly suitable for use in resource-limited, lower-biosafety-level primary laboratories.

## INTRODUCTION

As the fourth highest tuberculosis (TB) burden country in the world, China had an estimated 696,000 incident TB cases in 2024 (1). And the burden of drug-resistant TB cases, particularly rifampicin-resistant TB (RR-TB)/multidrug-resistant TB (MDR-TB), is especially severe, accounting for 29,000 cases (1). However, the treatment success rate in China was only 54% below the global average (59%), imposing a burden on healthcare resources (2). This situation is further exacerbated by the emergence of mono-isoniazid resistance (INHr), a pivotal precursor to MDR-TB, which is associated with alarmingly high treatment failure rates of 18–44% and severely compromises treatment efficacy (3–5). However, testing INHr is typically conducted only after rifampicin resistance is identified in China, resulting in the under-detection and inadequate management of INH-monoresistant TB cases (3). While fluoroquinolones (FQs) are essential for RR/MDR-TB treatment in short-course regimens, including bedaquiline (B), pretomanid (Pa), linezolid (L) (BPaL) or BPaLM (BPaL plus moxifloxacin), their widespread use for other infectious diseases has increased FQ resistance in *Mycobacterium tuberculosis* (MTB), thereby compromising treatment outcomes (6–8). Furthermore, the updated definition of pre-extensive drug-resistant tuberculosis (pre-XDR-TB) is MDR/RR-TB that is also resistant to any FQs (9). The severity of this issue is highlighted by a study in China, which found that 73.2% of MDR-TB isolates were FQ-resistant (10). Thus, early and rapid identification of INH and FQ resistance is critical for determining patient eligibility for optimal treatment regimens.

Current methods for detecting resistance to INH and FQs, such as culture-based phenotypic drug susceptibility testing (pDST) and line probe assays (LPAs), face several limitations. These include limited accessibility, as they are often confined to reference laboratories; prolonged turnaround times; complicated operation; and suboptimal sensitivity in smear-negative or paucibacillary specimens (11–13). Consequently, there is a need for simple, rapid, and accurate molecular diagnostics specifically designed to detect resistance-conferring mutations in genes associated with INH and FQ resistance.

Low-complexity automated nucleic acid amplification tests (LC-aNAATs) have emerged as a new generation of diagnostics designed to bridge this gap, which enable simultaneous detection of the relevant resistance mutations within 90 min and are suitable for intermediate and peripheral laboratory settings, greatly facilitating individualized treatment decisions for TB patients (14). Based on these advantages, the World Health Organization (WHO) conditionally recommends using LC-aNAATs as the initial test over culture-based DST for detecting INH and FQ resistance in confirmed pulmonary TB patients (14).

The iFIND INH/FQ assay (ROCGENE, China) is a LC-aNAAT that meets WHO criteria (14). It utilizes a fully integrated system for automated nucleic acid amplification to simultaneously identify resistance-associated mutations in the *inhA* and *katG* genes for INH, and *gyrA* gene for FQs. Similar to the iFIND TBR assay (15), which is a microfluidic-based, all-in-one test that integrates nucleic acid extraction, amplification, and detection for TB identification and rifampicin resistance determination within 85 min, the INH/FQ assay is designed to offer streamlined operation and rapid results in clinical settings. In this study, we aimed to systematically evaluate the analytical performance and diagnostic accuracy of the iFIND INH/FQ assay for detecting resistance of INH and FQs in MTB, and to assess its potential for clinical application.

## MATERIALS AND METHODS

### Clinical sputum specimens and bacterial strains

**Fig 1:**
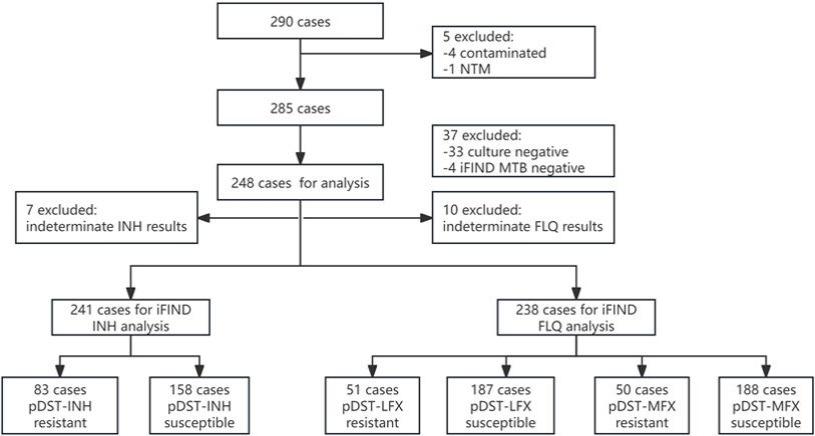
Flowchart of patient specimen enrollment and inclusion for final analysis.

A total of 290 frozen sputum specimens from suspected drug-resistant pulmonary TB cases, stored at the Chengde Center for Disease Control and Prevention laboratory, were initially enrolled in the study. After excluding culture-negative (*n* = 33), culture-contaminated (*n* = 4), nontuberculous mycobacteria (NTM) (*n* = 1), and iFIND-MTB negative samples (*n* = 4), 248 samples were included in the final analysis (Fig. 1).

### Mycobacterial culture and species identification

Mycobacterial isolates were initially cultured on Lowenstein-Jensen (L-J) medium. For species identification, the isolates were subcultured onto L-J medium containing 500 µg/mL paranitrobenzoic acid (PNB). Growth on the PNB-containing medium indicated NTM, whereas no growth confirmed *Mycobacterium tuberculosis* complex (MTBC).

### pDST

The proportion method on L-J solid medium was used for pDST. Critical concentrations of INH (0.2 µg/mL), levofloxacin (2 µg/mL), and moxifloxacin (1 µg/mL) were applied in accordance with WHO guidelines (16). A growth proportion of >1% on drug-containing medium compared to drug-free control was defined as resistant.

### iFIND assay

The iFIND INH/FQ assay was performed according to the manufacturer’s instructions. Briefly, approximately 1 mL of sputum was transferred to a sample tube and mixed with sputum processing solution at a 2:1 (vol/vol) ratio. The mixture was vortexed for 15–30 s and incubated for 15 min. Then, 2 mL of the processed sample was loaded into a cartridge for automated nucleic acid extraction, amplification, and detection. The assay reports semi-quantitative MTB load based on CT values: very low, low, medium, and high. Results for susceptibility are reported as “resistant,” “susceptible,” or “indeterminate.”

### Sequencing of discrepant results

Crude DNA was extracted from all isolates by resuspending mycobacteria in Tris-EDTA buffer, followed by heating at 100°C for 15–20 min and removal of cellular debris via centrifugation at 10,000 rpm for 10 min. Regions of the *gyrA*, *katG*, and *inhA* genes were amplified using the primers listed in Table 1 to clarify discordant results between the iFIND INH/FQ assay and pDST. PCR products were sent to Tsingke Co. (Beijing, China) for Sanger sequencing. The resulting DNA sequences were aligned with reference sequences from the MTB H37Rv strain using BioEdit Sequence Alignment Editor 7.1.3.

**TABLE 1:** Primers used for sequencing

Gene	The first round of amplification primers	Sequences (5′−3′)
*gyrA*	*gyrA*-F	CGGGTGCTCTATGCAATGTTCG
	*gyrA*-R	GGCCGTCGTAGTTAGGGATGAAAT
*katG*	*katG*-F	AGATGGGGCTGATCTACGTGA
	*katG*-R	GCTCATAGATCGGATCCACCC
*inhA*	*inhA*-F	ATACACCCGCAGCCAGGGCCTCG
	*inhA*-R	TTTCCTCGGTCATCCGCATG

Gene	The second round of amplification primers	Sequences (5′−3′)
*gyrA*	*gyrA*-F	GCTCTATGCAATGTTCGATTCC
	*gyrA*-R	GACTGTCTCCTCGTCGATTTC
*katG*	*katG*-F	CGGTCACACTTTCGGTAAGA
	*katG*-R	CGTCCTTGGCGGTGTATT
*inhA*	*inhA*-F	GCTGAGTCACACCGACAAA
	*inhA*-R	GTTGGACACCAGCACCTC

### Limit of detection (LOD)

The LOD was determined using MTB strain H37Rv spiked into negative sputum samples. In the initial evaluation, bacterial suspensions were diluted to final concentrations of 100, 10, and 1 CFU/mL, with five replicates per concentration. Based on preliminary results, further dilutions were prepared at concentrations of 20, 15, 8, 4, 2, and 0.5 CFU/mL, with 20 replicates per concentration, to more precisely determine the LOD.

### Detection of various mutant types

The assay’s ability to detect different mutations associated with INH and FQ resistance was evaluated using characterized strains. INH-related: *inhA* promoter (−15, −8), *katG* S315T, *inhA* −8/ahpC −12, *ahpC* −6; FQ-related: *gyrA* G88C, A90V, S91P, D94A, D94G, D94N, D94Y.

### Statistical analysis

Data were analyzed using SPSS version 20.0 (IBM, Chicago, IL). The LOD was calculated via probit regression analysis. Diagnostic accuracy, including sensitivity, specificity, positive prediction value (PPV), and negative predictive value (NPV), was described as point estimates with 95% confidence intervals (95% CIs). Agreement between the iFIND INH/FQ assay and pDST was assessed using Kappa analysis, with values interpreted as follows: 0.4–0.6 (moderate), 0.61–0.80 (substantial), and >0.80 (almost perfect).

## RESULTS

### LOD of the iFIND INH/FQ assay

**Fig 2:**
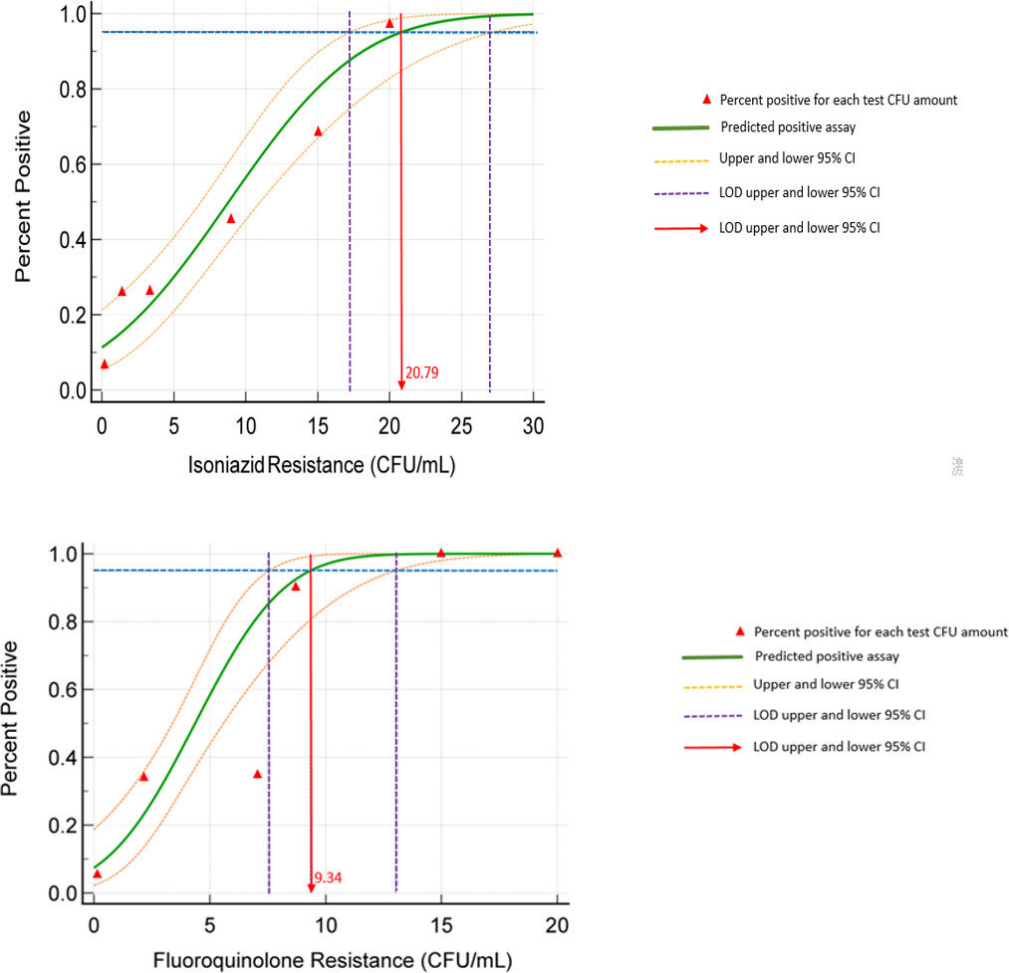
Probit regression analysis for determining the limit of detection of the iFIND INH/FQ assay.

In the initial evaluation phase, all replicates (5/5) at 100 CFU/mL and 80% (4/5) at 10 CFU/mL were accurately detected for both INH and FQ susceptibility (Table 2). At 1 CFU/mL, the detection rate was 60%. Subsequent probit regression analysis at the second phase determined the LOD to be 20.79 CFU/mL (95% CI: 15.13–39.91) for INH and 9.34 CFU/mL (95% CI: 7.54–13.10) for FQs (Table S1; Fig. 2). The iFIND assay successfully identified all targeted mutant genotypes associated with INH and FQ resistance, with the exception of one strain carrying an *ahpC* c.-6 mutation, which is not covered by the assay (Table S2).

**TABLE 2:** Limit of detection of the iFIND INH/FQ assay with H37Rv^*a*^

Replicate	Isoniazid			Fluoroquinolone		
	100 CFU/mL	10 CFU/mL	1 CFU/mL	100 CFU/mL	10 CFU/mL	1 CFU/mL
1	S	S	MTB Neg	S	S	MTB Neg
2	S	S	S	S	S	S
3	S	S	S	S	Ind	Ind
4	S	S	Ind	S	S	S
5	S	S	S	S	S	S
Detection rate	100%	100%	60%	100%	80%	60%

^*a*^ Ind, indeterminate; Neg, negative; S, susceptible.

### Diagnostic performance of iFIND

The iFIND assay produced indeterminate results in 2.8% (7/248) and 4.0% (10/248) of samples for INH and FQ resistance, respectively. Among the seven samples with indeterminate results for INH, three were graded as smear 1+, three as smear 2+, and one as smear 3+. Of the 10 samples with indeterminate results for FQ, 1 was graded as smear 4+, 1 as smear 3+, 2 as smear 2+, and 6 as smear 1+. These indeterminate calls occurred largely in samples with low bacterial loads (smear 1+ to 3+) (Table S3).

### Comparison of iFIND and pDST for detection of resistance

Compared to pDST, the iFIND assay demonstrated a sensitivity of 97.59% (95% CI: 91.63–99.34) and specificity of 98.10% (95% CI: 94.57–99.35) for INHr. For FQ resistance, sensitivity was 92.16% (95% CI: 81.50–96.91) for levofloxacin and 92.00% (95% CI: 81.16–96.85) for moxifloxacin, with specificities of 97.33% (93.89–98.85) and 96.81% (93.21–98.53), respectively (Table 3). Agreement between iFIND and pDST was high, with kappa values of 0.95 for INH, 0.89 for levofloxacin, and 0.88 for moxifloxacin.

**TABLE 3:** Detection capacity of the iFIND INH/FQ assay compared to phenotypic DST for detection of isoniazid and fluoroquinolone resistance^*a*^

Drug	iFIND	pDST		Sensitivity (%, 95% CI)	Specificity (%, 95% CI)	PPV (%, 95% CI)	NPV (%, 95% CI)	Kappa
		R	S					
INH	R	81	3	97.59 (91.63–99.34)	98.10 (94.57–99.35)	96.43 (90.02–98.78)	98.73 (95.47–99.65)	0.95
	S	2	155					
LFX	R	47	5	92.16 (81.50–96.91)	97.33 (93.89–98.85)	90.38 (79.39-95.82)	97.85 (94.60–99.16)	0.89
	S	4	182					
MOX	R	46	6	92.00 (81.16–96.85)	96.81 (93.21–98.53)	88.46 (77.03–94.60)	97.85 (94.60–99.16)	0.88
	S	4	182					

^*a*^ DST, drug susceptibility testing; INH, isoniazid; LFX, levofloxacin; MOX, moxifloxacin; NPV, negative predictive value; PPV, positive predictive value; R, resistant; S, susceptible.

### Inconsistent resistance results detected by iFIND and pDST

Sequencing analysis was performed on samples with discrepant results between iFIND and pDST. For INH, all five discordant samples were consistent with iFIND results, confirming the presence or absence of resistance-conferring mutations. Among nine FQ-discordant samples, sequencing supported iFIND in five cases (55.56%, 5/9) and pDST in four cases (Table 4).

**TABLE 4:** Sequencing results of discordant results between the iFIND assay and phenotypic drug susceptibility testing

Drug	Sample	iFIND	pDST	Sequencing	Mutant loci
Isoniazid					
	2,014	R	S	R	*inhA* −15 C→T
	2,078	R	S	R	*inhA* −15 C→T
	2,097	R	S	R	*inhA* −15 C→T
	1,027	S	R	S	Wild type
	3,046	S	R	S	Wild type
Fluoroquinolone					
	2,021	R	S	R	*gyrA* A90V
	2,042	R	S	R	*gyrA* D94G
	2,097	R	S	S	*gyrA* wild type
	2,120	R	S	R	*gyrA* S91P
	2,126	R	S	S	*gyrA* wild type
	2,022	S	R	R	*gyrA* S91P
	2,069	S	R	S	*gyrA* wild type
	1,094	S	R	R	*gyrA* D94A
	3,028	S	R	S	*gyrA* wild type

## DISCUSSION

Given the high resistance rates of INH and FQs in China, timely detection of resistance to these two drugs is particularly important. This study evaluated the performance of the iFIND INH/FQ LC-aNAAT assay for the rapid and simultaneous detection of MTB resistance to INH and FQs. The assay demonstrated LOD of 20.79 CFU/mL for INH and 9.34 CFU/mL for FQs, meeting the optimal LOD requirement (≤10^2^ CFU/mL) set by the WHO Target Product Profile (TPP) for sputum-based assays (17). The observed indeterminate rates of 2.8% for INH and 4.0% for FQ resistance in this study fall within the acceptable range (minimal requirements <10% and optimal <3%) according to WHO TPP recommendations for low-complexity automated NAATs (17). And the indeterminate results primarily occurred in samples with low bacterial loads, likely due to competition among PCR targets, which can suppress signals for certain mutations (18). To reduce indeterminate calls, future improvements could focus on optimizing nucleic acid extraction for low-biomass specimens, incorporating pre-amplification or isothermal methods to increase sensitivity. Further development may also explore digital PCR or droplet-based platforms for absolute quantification and better discrimination near the detection limit. Clinical validation in paucibacillary populations will be necessary to calibrate and verify this technical refinement.

In terms of diagnostic performance, this study showed that the sensitivity and specificity of the iFIND assay for detecting INHr was 97.6% and 98.1%, respectively. For FQs, the sensitivity was 92.2% (levofloxacin) and 92.0% (moxifloxacin), with specificities exceeding 96% for both. The assay showed a high degree of concordance with pDST, aligning with the requirements outlined in the WHO TPP for new TB diagnostic technologies at the peripheral level (17), which showed the minimal required sensitivity and specificity are set at >90% and ≥98%, respectively, for detecting INH and FQ resistance, and with optimal targets aiming even higher (>95% sensitivity) by WHO. Besides, this result is highly comparable to the published performance of the Xpert MTB/XDR assay (which detects resistance to INH, FQs, and second-line injectable agents), which reports a sensitivity of approximately 94% for INH and 93% for FQs and a specificity of 98% (19). In addition to diagnostic performance, a similar workflow was observed between these two methods with 15-min specimen processing prior to loading. However, the total turnaround time for the iFIND assay (≤150  min) is longer than that of Xpert MTB/XDR (≈90 min), which primarily reflects the distinct amplification chemistries and detection modules employed by the two platforms. More importantly, for samples with discrepant results between iFIND and pDST, sequencing mostly confirmed the accuracy of the iFIND results, highlighting the potential advantage of molecular detection in identifying resistance-conferring mutations compared to pDST. Compared to LPAs, the iFIND assay offers the decisive advantages of automation and speed while maintaining similarly high sensitivity and specificity (20, 21). Compared to emerging WHO-recommended targeted sequencing technologies (22, 23), while iFIND’s mutation coverage is not as broad (e.g., the *ahpC* c.-6 mutation in this study was not covered), its strengths lie in its extremely fast turnaround time, lower data analysis complexity, and cost. Though sequencing is the standard for discovering novel mutations and investigating resistance mechanisms, its technical requirements, turnaround time, and cost currently make it more suitable as a complementary or confirmatory tool in central reference laboratories rather than a first-line rapid screening test. iFIND is precisely positioned for first-line rapid screening, covering the most common clinically relevant mutations (e.g., *katG* S315T, *inhA* promoter, and *gyrA* 90/94), which is sufficient for the vast majority of clinical scenarios. And future versions of iFIND could include additional mutations such as ahpC c.-6 to further improve sensitivity, especially in settings where less common resistance mechanisms may be emerging.

The WHO TPP also emphasizes that the ideal diagnostic tool for detecting XDR should be rapid (results in <6 h), simple to operate (minimal requirements of manual preparation of samples ≤5 steps, optimal ≤1 step), and deployable in low-level laboratories (peripheral laboratories) to guide timely and effective treatment decisions (17). As a LC-aNAAT, the iFIND assay achieves the goal of simultaneously detecting INH and FQ resistance in approximately 150 min, perfectly meeting the TPP’s core requirements for “rapid” and “simple operation”. Its fully integrated system minimizes manual steps and contamination risk, making it highly suitable for use in resource-limited, lower-biosafety-level primary laboratories.

Nevertheless, this study has certain limitations. The samples used in this study were from a single center and were frozen specimens, which may introduce selection bias and risks associated with sample degradation. Therefore, future multi-center, prospective studies that include fresh clinical specimens are needed to better simulate real-world testing conditions. Furthermore, as with all molecular diagnostics, the performance of iFIND is contingent upon the prevalence of the targeted mutations, and its real-world applicability in diverse epidemiological settings requires further validation through prospective studies.

In conclusion, the iFIND INH/FQ assay is a highly reliable LC-aNAATs suitable for rapid and simultaneous detection of INH and FQ resistance. Its implementation in peripheral laboratories can enhance TB case management by enabling timely, decentralized resistance testing, supporting earlier appropriate treatment, and strengthening local TB control.

## Data Availability

Data supporting the results can be found in this paper. The data sets generated and analyzed during the current study are available from the corresponding author, Yanlin Zhao (zhaoyl@chinacdc.cn) on reasonable request.

## SUPPLEMENTAL MATERIAL

Supplemental tables spectrum.03743-25-s0001.docx Tables S1 to S3.

ASM does not own the copyrights to Supplemental Material that may be linked to, or accessed through, an article. The authors have granted ASM a non-exclusive, world-wide license to publish the Supplemental Material files. Please contact the corresponding author directly for reuse.
