# Supplementary material for: iFIND INH/FQ: a LC-aNAAT assay for rapid simultaneous detection of isoniazid and fluoroquinolone resistance in Mycobacterium tuberculosis
Source: Microbiol Spectr. 2026 Feb 6;14(3):e03743-25. doi: 10.1128/spectrum.03743-25 (PMC12955496; doi:10.1128/spectrum.03743-25)
Supplement: Supplemental tables — Tables S1 to S3. [file spectrum.03743-25-s0001.docx]

Table S1. Comprehensive determination of the limit of detection for the iFIND INH/FQ Assay

| Concentration (CFU/mL) | Replicate | Positive | |
| --- | --- | --- | --- |
|  |  | Isoniazid | Fluoroquinolone |
| 20 | 20 | 20 (100) | 20 (100) |
| 15 | 20 | 13 | 20 |
| 8 | 20 | 11 | 18 |
| 4 | 20 | 5 | 7 |
| 2 | 20 | 5 | 7 |
| 0.5 | 20 | 1 | 1 |

Table S2. Detection capacity of the iFIND INH/FQ assay for various mutant types associated with isoniazid and fluoroquinolone resistance

| Mutation Sites | No. strains | Isoniazid | | Fluoroquinolone | |
| --- | --- | --- | --- | --- | --- |
|  |  | Susceptible | Resistant | Susceptible | Resistant |
| inhA -15 | 4 | 0 | 4 | NA | NA |
| katG S315T | 9 | 0 | 4 | NA | NA |
| katG S315T / inhA -15 | 2 | 0 | 2 | NA | NA |
| inhA -8T>A | 1 | 0 | 1 | NA | NA |
| inhA -8 / ahpC -12 | 1 | 0 | 1 | NA | NA |
| ahpC -6 | 1 | 1 | 0 | NA | NA |
| gyrA G88C | 1 | NA | NA | 0 | 1 |
| gyrA A90V | 1 | NA | NA | 0 | 1 |
| gyrA S91P | 1 | NA | NA | 0 | 1 |
| gyrA D94A | 1 | NA | NA | 0 | 1 |
| gyrA D94G | 1 | NA | NA | 0 | 1 |
| gyrA D94N | 1 | NA | NA | 0 | 1 |
| gyrA D94Y | 1 | NA | NA | 0 | 1 |

NA: Not applicable

Table S3 Indeterminate results of the iFIND INH/FQ assay

| Drug | Smear results | Number |
| --- | --- | --- |
| Isoniazid (N=7) | 1+ | 3 |
|  | 2+ | 3 |
|  | 3+ | 1 |
| Fluoroquinolone (N=10) | 1+ | 6 |
|  | 2+ | 2 |
|  | 3+ | 1 |
|  | 4+ | 1 |
